# Stigma and utilization of treatment for adolescent perinatal depression in Ibadan Nigeria

**DOI:** 10.1186/s12884-020-02970-4

**Published:** 2020-05-14

**Authors:** Lola Kola, Ian M. Bennett, Amritha Bhat, Olatunde O. Ayinde, Bibilola D. Oladeji, Dolapo Abiona, Jibril Abdumalik, Neda Faregh, Pamela Y. Collins, Oye Gureje

**Affiliations:** 1 Department of Psychiatry, College of Medicine, University of Ibadan, Ibadan, Nigeria; 2 Department of Sociology and Psychology, Faculty of Social Sciences, Lead City University, Ibadan, Nigeria; 3 Department of Psychiatry College of Medicine, WHO Collaborating Centre for Research and Training in Mental Health, Neurosciences and Drug and Alcohol Abuse, University of Ibadan, Ibadan, Nigeria; 4 Departments of Family Medicine, University of Washington, Seattle, USA; 5 Psychiatry and Behavioral Science, University of Washington, Seattle, USA; 6 Global Health, University of Washington, Seattle, USA; 7 Department of Psychology, Carleton University, Ottawa, Ontario Canada

**Keywords:** Perinatal depression, Adolescent, Pregnancy, Stigma, Health care utilization, Resilience, Low- and middle-income country

## Abstract

**Background:**

Depression is a common and severe disorder among low-income adolescent mothers in low-and middle-income countries where resources for treatment are limited. We wished to identify factors influencing health service utilization for adolescent perinatal depression, in Nigeria to inform new strategies of care delivery.

**Methods:**

Focus Group Discussions (FGDs) were conducted among purposively selected low-income young mothers (with medical histories of adolescent perinatal depression), and separately with primary care clinicians treating this condition in Ibadan, Nigeria. Participants from this community-based study were from the database of respondents who participated in a previous randomized control trial (RCT) conducted between 2014 and 2016 in 28 primary health care facilities in the 11 Local government areas in Ibadan. Semi-structured interview guides, framed by themes of the Behavioral Model for Vulnerable Populations, was developed to obtain views of participants on the factors that promote or hinder help-seeking and engagement (see additional files 1 & 2). FGDs were conducted, and saturation of themes was achieved after discussions with six groups. Transcripts were analyzed using content analysis.

**Results:**

A total of 42 participants, 17 mothers (who were adolescents at the time of the RCT), and 25 care providers participated in 6 FGDs. The availability of care for perinatal depression at the primary care level was an important enabling factor in healthcare utilization for the adolescents. Perceived health benefits of treatment received for perinatal depression were strong motivation for service use. Significant stigma and negative stereotypes expressed by care providers towards adolescent pregnancy and perinatal depression were obstacles to care. However, individual patient resilience was a major enabling factor, facilitating service engagement. Providers trained in the management of perinatal depression were perceived to deliver more tolerant and supportive care that adolescent mothers valued.

**Conclusions:**

Participants identified unsupportive and stigmatizing clinic environments towards pregnant and parenting adolescents as significant barriers to accessing available care. Interventions to reduce stigma among healthcare providers may improve services for this vulnerable population.

## Background

Maternal health care for adolescents is complex and involves a range of psychosocial issues while also requiring attention to both the developmental needs of the child-parents and medical problems related to the pregnancy [28]. Approximately 95% of all births to girls under age 18 occur in low- and middle -income countries (LMICs), where supporting infrastructure for healthcare is limited (UNFPA, 2013). Perinatal depression (PD), occurring during pregnancy and in the year postpartum, is common among adolescent mothers than older mothers of childbearing age [4, 24]. The occurrence of PD among adolescents complicates their unique age-and development-related challenges and thus demands services attuned to the unique needs of this age group. Evidence from Nigeria suggests that children of adolescent mothers with depression have poorer outcomes [21]. However, Nigeria, like other low resource nations of the region, lacks adequate numbers of trained care providers as well as appropriate infrastructure to support high quality primary maternity care for adolescents [31].

Adolescent mothers in LMICs have reported a high degree of stigmatizing attitudes regarding adolescent sexual activity from healthcare providers, which reflects broader social and cultural forces to healthcare participation [3, 14, 20, 29]. Self-stigma and younger age have been associated with mental help-seeking among young people [12], and perinatal adolescents with depression, have reported additional experiences of social stigma from family members, partners, and people in their social environments, as barriers to health care utilization [13]. Stigma is a fundamental cause of health inequities [10] and stigmatizing attitudes from clinic staff may lead to dissatisfaction with the health system, reduce the likelihood of help-seeking, and eventually compromise maternal and infant health outcomes [11, 18, 30]. These finding suggest that programs addressing perinatal depression in adolescents may need to address stigma towards their pregnancy to be effective.

We wished to explore factors associated with care for adolescents with perinatal depression in an urban setting in Nigeria and identify potential interventions to improve care utilization. In this study, we utilized the Behavioral Model for Vulnerable Populations [7], which provides a systematic framework for exploring how vulnerable individuals engage with the health service. Specifically, our research question was, how does predisposing, enabling, and need factors impact the health service utilization experiences of perinatal adolescents with depression?

## Methods

### Subjects and study setting

This qualitative study was carried out in Ibadan, Southwest Nigeria. Using purposive sampling, we selected participants from our database of respondents from 28 primary health care facilities in the 11 Local government areas in Ibadan, who participated in the treatment arm of a previous randomized control trial (RCT) conducted between 2014 and 2016 [8]. We did a total of 6 Focus Group Discussions (FGDs) with three mothers groups (*n* = 17), and three primary care providers groups (*n* = 25) between 6th March and 29th April 2018. In the RCT, our team trained non-specialist maternal and child health clinicians (MCHC) to deliver psychosocial interventions for perinatal depression using the Nigerian adapted WHO mhGAP intervention guide (mhGAP-IG) [9]. The 17 mothers who were adolescents when the RCT was conducted, were no longer adolescents at the time of the FGDs. Also, while they were diagnosed with moderate to severe perinatal depression during the RCT, all had recovered from their depression at the time of the present study. The number of participants per group ranged between 5 and 9, as indicated in Table 1. All participants were above the age of 18 years at the time of the interview and gave written informed consent to participate in the study. The mother groups were conducted separately from the care provider groups. Incentives of approximately $15 united states dollars were given to all participants. The study was approved by the University of Ibadan/University College Hospital Institutional ethics review committee.

**Table 1:** Study participants according to groups

Women group 1 (*n* = 5)	those who completed 8 sessions of treatment
Women group 2 (*n* = 7)	those who completed 6 sessions of treatment
Women group3 (*n* = 5)	those who completed ≤ 2
Provider group 1 (*n* = 8)	Heads of ANC facilities who are midwives
Provider group 2 (*n* = 9)	Intermediate level clinicians
Provider group 3 (*n* = 8)	A mix of the various cadres of clinicians including junior, intermediate and heads of facilities

### Semi-structured interview guide

Guided by the themes of the behavioral model for vulnerable populations [7], authors OG and LK designed a semi-structured question guide, to explore predisposing, enabling, need and outcome factors of health care use of adolescents with PD. Their health behaviors were also investigated (see Table 2). Our use of a semi-structured design removed the restriction implicit in the use of closed-ended questions [22] and allowed for the use of probes for necessary explorations. The guide questions draw on the experiences of participants during the trial and form the basis of this paper. The interview guide was initially written in English and later translated to the Yoruba language. The Yoruba version of the interview guide was necessary to ensure a better understanding of the concept under study by participants.

**Table 2:** Conceptual themes and definitions (derived from the Behavioral Model for Vulnerable Populations)

Conceptual themes	Definitions
Predisposing factors	Demographic, health beliefs and other factors prompting their utilization of health facilities
Enabling factors	Personal, family and health care resources available to the women during their pregnancy
Need factors	Perceived and evaluated health
Health behavior	Personal health practices
Outcome factors	Satisfaction with care and other effects of service use

### Data collection

Participants were contacted on the phone and invited to participate in the FGDs. As presented in Table 1, the mothers were selected according to their participation in the RCT specifically, to capture variations of experiences of care, from mothers who dropped out of the RCT treatment at various stages and those that completed treatment. Likewise, the care providers were identified based on their years of experience in order to obtain a variety of views based on years of practice. The FGD sessions were conducted in a seminar room at the University of Ibadan within a period of 3 weeks. The choice of venue was influenced by considerations of participant relaxation and safety, as well as its centrality to public transportation. LK, facilitated the FGDs and DA a research supervisor with 4 years of experience in qualitative study was the timekeeper/notetaker. In all the group discussions, LK declared to participants that she was not from a medical background, and this proved valuable because participants were able to let down their guard and relax during the discussions. (making the data rich and minimizing bias). Data collection and data analysis occurred simultaneously to ensure that all themes were exhaustively explored. FGDs were conducted and saturation of themes was achieved after the sixth group. The FGDs lasted between 70 and 90 min, and all were tape-recorded and transcribed. Two bilingual interpreters translated from Yoruba to English individually, and then came together to resolved discrepancies through consensus.

### Conceptual models

**Fig. 1:**
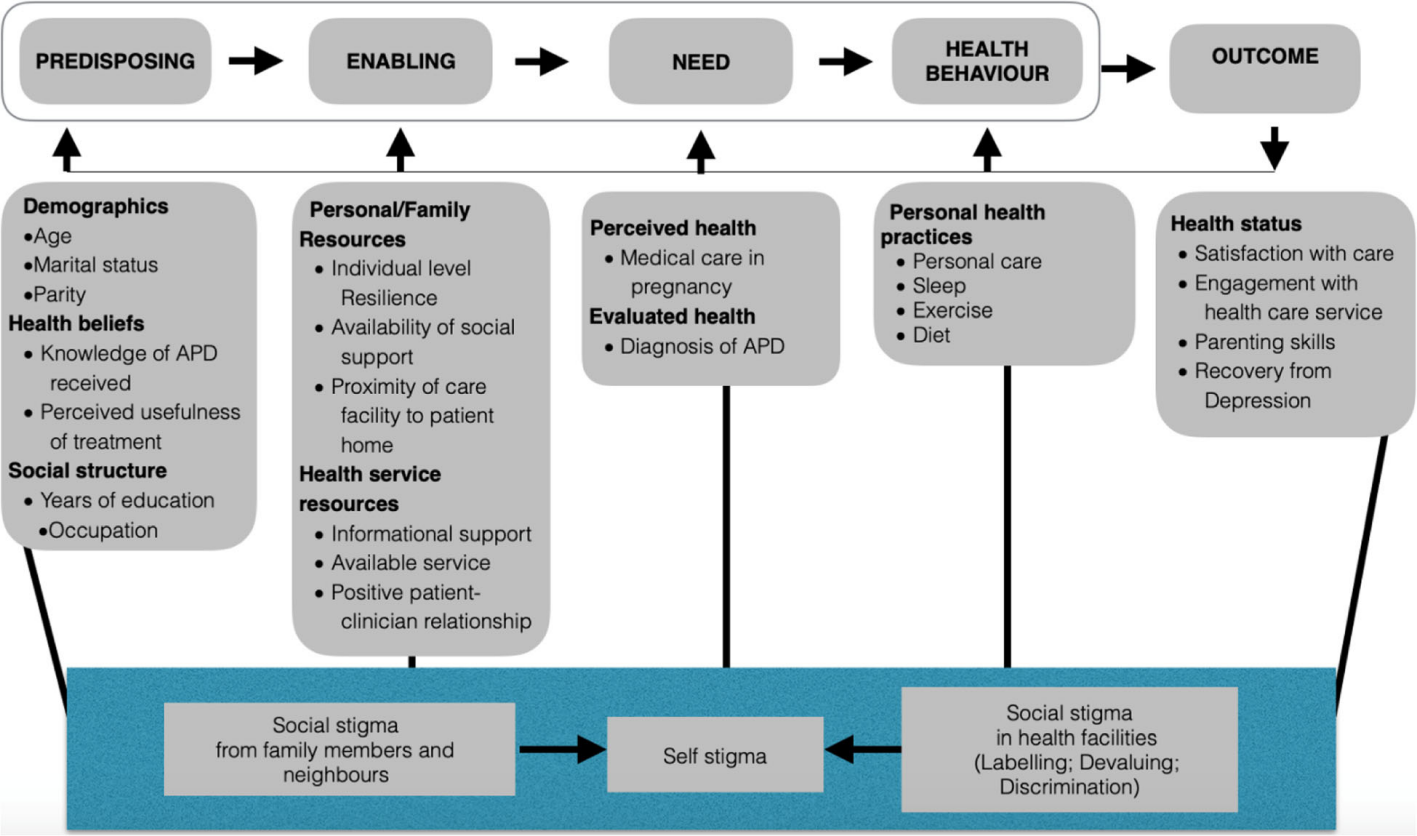
Behavioural model of health service use by adolescents with perinatal depression

The Behavioral Model for Vulnerable Populations (Fig. 1 [7];) guided the exploration during the FGDs and the analysis. This was based on the assumption that several factors determine the use of services [1, 2]. In Table 2, we present the main features of this model.

### Data analysis

All transcripts were analyzed using content analysis of themes that emerged from the FGDs [5]. Interpretation of the text data was guided by our conceptual model. This was achieved through the systematic classification process of coding and avoidance of preconceived categories [15]. This allowed for inductive categorization development according to themes and the emergence of insights into the data [15, 19]. Initial primary codes were created by LK from the interview guide under the thematic headings and then applied to the transcriptions. LK and DA independently coded the texts that reflect the utilization pattern of the then adolescents. Dissenting codes were resolved through consensus. Emerging recurrent themes were managed to avoid repetition. LK who holds a Ph.D. in medical sociology, and currently a senior research fellow, and an adjunct associate professor of Sociology, with years of experience in the conduct and analysis of qualitative interviews carried out the data analysis. Her social science background influenced the entire process of analysis to ensure objective in reporting of study findings. The interpretation of results carried out by LK, IB, DA, AB, PYC and OG (who had various background), thus allowing for the unbiased presentation of the data findings. This analysis led to the development of a descriptive account focused on identifying key variables and bringing out the range and diversity for each subject heading [23].

## Results

We contacted 24 mothers, and while all accepted our invitation to participate in the FGDs, only a total of seven teen (71%), actually took part in the interviews. For the care provider groups, we contacted twenty-six (26) persons on the phone, out of which one, declined our invitation to participate at the last minute because of an emergency work commitment resulting in 96% participation. The care providers were comprised of 3 men whose views did not differ from those of the women and neither was there any obvious gender tension during the FGDs. The results are organized below according to the constituent factors of the Behavioral Model.

### Predisposing factors

The ***demographic details*** of the participants are shown in Table 3. The mean age of the mothers was 22 ± 1.1 years, and their mean years of education (highest level of educational attainment) was 11.2 ± 1.7 years. Most of the mothers (11) and their partners (13) were low-income earners. The Maternal and Child Health care (MCHC) providers had a mean age of 49 ± 4.8 years; the majority were female and married. All the adolescents reported that care for their pregnancies was the reason they made their first visits to the clinics. Analysis of the FGD transcripts revealed some key findings to support, illustrate and illuminate the ideas and experiences of participants [25].

**Table 3:** Demographic characteristics of FGD Participants (*N* = 42)

***Women participants***	
Variable	N (%)
**Marital status**	
Married	14 (82.4)
Cohabiting	3(17.6)
**No of Children**	Total
**1**	3 (17.6)
**2**	13 (76.5)
**3**	1 (5.9)
**None**	0 (0)
**Occupation**	Participants
Apprentice	1 (5.9)
Artisans	11 (64.7)
Traders	4 (23.5)
Housewife/unemployed	1 (5.9)
	Mean + SD
**Age of women participants**	22 ± 1.1
**Years of education of women participants**	11.2 + 1.7
***Care provider participants***	
**Variable**	N (%)
**Sex of care providers**	
Male	3 (12.0)
Female	22 (88.0)
Total	25 (100.0)
**Marital status**	
Married	25 (100.0)
	Mean + SD
**Age of care provider participants**	49 ± 4.8

### Health beliefs

All FGD participants (mothers and providers), reported that the primary reason the mothers attended clinic appointments for the treatment of perinatal depression was the perceived usefulness of the care received for the condition. All of the mothers reported no ***knowledge of having depression*** before they visited the antenatal clinic (ANC), where they were recruited into the trial following screening for depression. Their reports specifically showed they lacked the awareness of the nature of depression and its symptoms, which they mostly thought to be normal features of pregnancy.

### Enabling factors

We present the enabling factors of healthcare utilization of the FGD participants with a focus on the mothers’ experiences as they interacted with service and clinic staff. The women reported the availability of care for perinatal at the primary care level was an essential enabling factor in healthcare utilization for the adolescents.

*“I met matron [name] and she gave me hope. She told me I had a sickness of the mind that made me sad… and that I will get better with time and I did. I like her a lot*” (FGD Mothers group 1).

The mothers described how they overcame self and social stigma relating to their early pregnancy to access available care. They reported that increased visits to the health facility resulted in improved clinical conditions, which encouraged their continuing use of healthcare services.

*“…I was in a bad state when I went to the clinic… I thought my life was over. However, the more I visited the clinic and talked to the matron, the better I felt”.* (FGD, Mothers group 1).

### Social support

Many of the mothers reported low social support which was however still an enabler to use of service.

“My mother *showed me very little support and was not happy about my pregnancy…but she would make sure I go the clinic…”.* (FGD, Mothers group 1)

“*My mother followed me to the clinic only once”* (FGD, Mothers group 3)

Providers reported as very essential, the mhGAP training they received on the management of perinatal depression before the trial, which quipped them with the knowledge to manage the condition. The training not withstanding about half of the providers expressed openly disparaging views about the difficulties of managing adolescent mothers, and endorsed a justification for the low social support they received from relatives during their pregnancies.

*“Many of these girls are irresponsible and promiscuous and do not listen to parents …it is no wonder that they receive very little support from relatives. They need to learn the hard way.”*

(FGD, Care provider group 2)

The ***informational support*** the mothers received from the care providers was rated as very helpful to their understanding of the symptoms of depression. Though majority of the mothers expressed appreciation for available health care services for perinatal depression at the primary care clinic level, many reported stigmatizing attitudes of other clinic staff and junior nurses (who typically had no training in the management of perinatal depression, and were not part of the previous trial). These junior nurses were usually in charge of conducting physical examinations of pregnant mothers at the primary care clinics, before they are seen by more experienced nurses. Stigma experienced by the adolescent women, described as being sometimes subtle, was manifested through negative attitudes and behaviors. Examples included receiving sharp answers to questions asked during a physical examination, or overt statements of negative stereotypes such as telling a patient she was too young to get pregnant. The mothers reported passive and active strategies of coping with the negative attitudes of such the clinic staff. In the excerpt below, a participant recounted a case with a junior nurse who was not part of the RCT.

*“I had a bad experience one day with a nurse [name]. She was the one that checks everyone to see if the babies are breathing, nurse [name] was always rude to young people. She told me that I got pregnant when my mates were in school… I felt very worthless …, yet I answered back and told her she was a bad person. I refused to let her attend to me after that day”.* (FGD, Mothers group 2).

Others recalled how they ignored negative remarks to continued clinic visits despite the unpleasantness of some staff.

*“I continued going to see matron and didn’t pay too much attention to discouraging comments from people…”.* (FGD, Mothers group 3).

*“As for me, I just faced what I went to the clinic for…”.* (FGD, Mothers group 3).

*“I went to the clinic only when i am sure [the] matron was on duty”.* (FGD, Mothers group 1).

The care provider groups likewise pointed to difficulties with clinic structure, particularly on ANC days when the mothers made appointments for both routine perinatal and depression care to reduce the frequency of travel to the clinics. Many of the mothers reported ANC days as difficult, with long waiting times. Patients expressed dissatisfaction with the practice of being called only once, or not loud enough when it is their turn to be attended to by the clinician. This practice often led to the patient missing their allotted positions in the queue.

*“Going for ANC was a big problem. The lines were long and the record clerks called our names only once. If you missed when your name was called, then you might have to wait a long time…”.* (FGD, Mothers group 3).

Although the reports of the providers corroborated systemic care delivery shortcomings relating to human resource limitations, they attributed aspects of poor care quality to the difficulties of working with adolescents.

*“Those young girls are very rude and difficult to attend to. They are very disrespectful”.* (Care provider group 1).

Though all the mothers rated the perceived usefulness of treatment they received for depression as high, the FGDs with providers reflected a lack of sympathy and frustration in caring for adolescents with PD. They described adolescents with PD as more “annoying” because of the symptoms of depression that made them socially withdrawn.

However, despite significant examples of stigmatizing behaviors, the mothers reported *positive patient and clinician relationships with the trained care providers who managed their depression (that is those trained during our RCT).*

### Need factors

The mothers reported perceived heightened need to continue in treatment after the diagnosis of their depression. This was facilitated by the support they received from their MCHC providers despite some of their unpleasant experiences they reported from the clinics.

*“The matron that took care of me was very nice to me…”.* (FGD, Mothers group 3).

### Health behavior

The providers’ accounts depicted many adolescents in a degrading manner as having poor personal hygiene practices that sometimes made physical examination unpleasant. One care provider noted,

*“Many of those girls are very dirty. When you want to examine them, they smell”*. (FGD Care provider group 3).

The mothers reported relatively few recommended health practices, although health talks were given to them regularly by their providers. About half of the mothers indicated being aware of the need to have adequate ***sleep***. Four reported walking about an hour per day not intentionally for ***exercise*** but mainly to run errands. Many reported having very few food choices as a result of poor social and economic circumstances. The MCHC clinicians in their account also related that generally, adolescents have poor health practices because of lack of autonomy:

“…*it can be difficult for someone young to enforce anything at home because they are not autonomous.”*. *(FGD Care Provider Group 1)*

### Outcome following the receipt of care

There was consensus across the mothers’ FGD groups on ***satisfaction with the care, and engagement with health care services.*** All mothers interviewed had ***recovered from their depression***.

## Discussion

In this study, we explored factors that may promote or hinder the use of health care services by adolescents experiencing perinatal depression. We used the Behavioral Model for Vulnerable Populations, that allows for a systematic exploration of developmental and contextual issues that may be of particular relevance when considering adolescents’ engagement with the health service in a resource-constrained setting. In health care services provided to support the mental health of adolescent mothers with depression, we identified evidence of stigma among providers, which had the potential to reduce the service use of perinatal adolescents.

The main predisposing factor to engaging with service by the adolescents was health beliefs about pregnancy and the perceived benefits of health services [26]. The mothers were generally unaware of their depression before being screened in the clinic and found to be depressed therefore, the availability of services for perinatal depression in primary care enabled them receive care for the condition. The subsequent interaction with the providers and informational support given to them led to an informed health belief about depression and the need to receive care for the condition.

The service environment was often discouraging, with long queues and stigmatizing attitude by many of the providers. In the clinic, the stigma was about both their early pregnancy and their depression. Untrained clinic staff labeled, devalued, stereotyped, and degraded young mothers, thereby undermining the primary goal of the provision of high-quality patient-centered health services by healthcare professionals. According to previous studies, social stigma towards adolescent pregnancy (experienced at home and at the clinics by the women) is rooted in cultural values and negative stereotypes which translate adolescent pregnancy into a stigmatized identity [6]. Training received by care providers in the management of depression prior to our RCT, was helpful to their engagement with the young mothers to manage the condition, but not entirely on their attitude towards adolescent pregnancy. We found that family resources like parental support were often absent for young mothers, and family members conveyed disapproval for the unplanned and unwanted pregnancies by providing little support for young girls during the perinatal period.

However, despite the vulnerability and demanding personal circumstances of many adolescents, they employed different coping strategies to use available health care services and deal with social stigma at the care facilities. Individual resilience was an important enabling factor that facilitated their continued use of treatment facilities [16, 17, 27].

### Limitations

The use of a semi structured interview guide in this study has the potential weakness of omitting some important and salient areas. This limitation is mitigated by the benefits of framing interviews and analyses based on a robust model of health services use. We also allowed for novel themes to emerge beyond those framed by our conceptual model. This limitation notwithstanding, our study presents a set of obstacles and opportunities to target health service interventions to improve the utilization of care by adolescents with perinatal depression.

## Conclusion

This study identified predisposing, enabling, and hindering factors to service utilization by adolescent mothers. We found that even though negative attitudes at home and in the treatment facilities were potential barriers, nevertheless adolescent mothers displayed a commendable level of resilience to cope with both their adverse social circumstances and the less than optimal service environment. We also found evidence that training care providers in the management of perinatal depression had an impact in their engagement with depressed young mothers. Our results highlight the need for improvement and more supportive health care delivery environments for pregnant adolescents to reduce barriers to care.

## Supplementary information

**Additional file 1.** Responding to the challenge of adolescent Perinatal Depression (RAPID) Focus Group Discussions for Adolescent mothers. Semi-structured interview guides, framed by themes of the Behavioral Model for Vulnerable Populations, developed to obtain views of participants on the factors that promote or hinder help-seeking and engagement of young mothers.

**Additional file 2.** Responding to the challenge of adolescent Perinatal Depression (RAPID) Focus Group Discussions for Care providers. Semi-structured interview guides, framed by themes of the Behavioral Model for Vulnerable Populations, developed to obtain views of participants on the factors that promote or hinder help-seeking and engagement of young mothers.

## Data Availability

The data that support the findings of this study are available from the corresponding author but restrictions apply to the availability of these data, which were used under license for the current study, and so are not publicly available. Data are however available from the authors upon reasonable request and with permission of the corresponding author.

## Abbreviations

PD: Perinatal Depression
RCT: Randomized Control Trial
LMICs: Low and Middle Income Countries
ANC: Antenatal Clinic
FGD: Focus Group Discussion
MCHC: Maternal and Child Health Care Clinician
