# Supplementary material for: Stigma and utilization of treatment for adolescent perinatal depression in Ibadan Nigeria
Source: BMC Pregnancy Childbirth. 2020 May 14;20:294. doi: 10.1186/s12884-020-02970-4 (PMC7226964; doi:10.1186/s12884-020-02970-4)
Supplement: Supplementary file 1 — Additional file 1. Responding to the challenge of adolescent Perinatal Depression (RAPID) Focus Group Discussions for Adolescent mothers. Semi-structured interview guides, framed by themes of the Behavioral Model for Vulnerable Populations, developed to obtain views of participants on the factors that promote or hinder help-seeking and engagement of young mothers. [file 12884_2020_2970_MOESM1_ESM.docx]

**Responding to the challenge of adolescent Perinatal Depression (RAPID)**

**Focus Group Discussions for Adolescent mothers**

**Introduction:**

I thank you all for coming to this group discussion today. I know that you have many things you need to do, and I really appreciate your taking the time to join other participants and me. As you were told on the phone, you have been invited because you participated in the EXPONATE trial where primary care providers delivered you care for your then depression in pregnancy. We are aware that the experiences of young women like you might have been different from the experiences of older pregnant women. We are also aware that as younger women, you might have had more health care needs than is currently available in primary care. That is why we have designed a new project titled RAPID to address the health care needs of young mothers specifically. To be able to design adequate care for young mothers on the RAPID project, it is important we learn from the experiences of young people like you who have recently experienced such care. If you agree with what I have said so far, I will like to give you more details about your participation, and I will also require you to sign on the consent document we will be providing you as a documentation of your agreement. Also, please note that I will be recording the interview so that I am sure not to make any mistakes in what you say and to make it easier for me to check that I am accurate.

**[The Facilitator should read the information details, on the inform consent form to each participant separately. All participants that agree to participate in the study should sign the consent document].**

*Predisposing factors*

**Facilitator to fill the basic demographic forms for participants who have given written consent to participants,**

Now that you have all agreed to participate, is it okay to start?

*Health beliefs.*

**1.** I will like to start with the reasons you went to the clinics for treatment. Many reasons make a woman attend the clinic during pregnancy. Can you please tell me the reasons you had to go to the clinic when you were pregnant?

Facilitator to probe for

- - Reasons for first clinic visits
  - Reasons relating to depressive symptoms patients might have experienced during the period.

2. You all had different symptoms of depression such as sadness, low of interest, low energy, inability to sleep, loss of appetite, and other symptoms that lasted for more than two weeks before you were diagnosed with depression during pregnancy. I will want to know if and how the treatment you were offered for depression by your care provider during the EXPONATE study addressed these symptoms

Facilitator to probe for

- - Aspects of the intervention that the patient found useful and unuseful (psychoeducation, problem-solving treatment, parenting skills)

*Enabling Factors.*

3. The next area for our discussion is keeping clinic appointments. There might be reasons that make young women want to or not want to attend clinic appointments during pregnancy. I would like us to know your views on these reasons as they relate to your own experiences.

Facilitator probe

- - Reasons relating to clinic appointments for depression treatment
  - Experiences with care and interactions with service and clinic staff

4. I will like to know how much support you had during your pregnancies in relation to clinic attendance for your depression.

Facilitator probe

- - Emotional and instrumental and informational support from family members, social environment
  - Did you get any kind of such support from clinic staff

*Quality of care and structural support in primary care*

5. Did you experience any specific difficulties in treatment delivered to you by the care provider?

Facilitator probe

- - Treatment-related to aspects of EXPONATE care

6. Did you require any need for additional support? (from other care providers) and how available was this support?

Probe for

- - your interactions with the clinic staff during receipt of treatment for depression
  - Probe for positive and negative results of such interactions

*Health behavior.*

7. The next question is about your health behavior during pregnancy. I will like to know what types of health behaviors you were aware of and how you got to know about these.

Probe for

- - average duration of any physical exercise mentioned
  - Consumption of healthy and adequate diet
  - Time for adequate rest and sleep

*Outcome following the receipt of care.*

8. I will like to know how satisfied you were with the care you received from the clinic for your depression during pregnancy. I am interested in knowing how your interactions with your care providers affected your recovery
