# Supplementary material for: Stigma and utilization of treatment for adolescent perinatal depression in Ibadan Nigeria
Source: BMC Pregnancy Childbirth. 2020 May 14;20:294. doi: 10.1186/s12884-020-02970-4 (PMC7226964; doi:10.1186/s12884-020-02970-4)
Supplement: Supplementary file 2 — Additional file 2. Responding to the challenge of adolescent Perinatal Depression (RAPID) Focus Group Discussions for Care providers. Semi-structured interview guides, framed by themes of the Behavioral Model for Vulnerable Populations, developed to obtain views of participants on the factors that promote or hinder help-seeking and engagement of young mothers. [file 12884_2020_2970_MOESM2_ESM.docx]

**Responding to the challenge of adolescent Perinatal Depression (RAPID)**

**Focus Group Discussions for Care providers**

**Introduction:**

I thank you all for coming to this group discussion today. I know that you have many things you need to do, and I appreciate your taking the time to join other participants and me. As you were told on the phone, you have been invited because you participated in the EXPONATE trial. As care primary care providers on trial, you delivered care to adolescents and older women with perinatal depression. We are aware that delivering care to young women might have been different from care to older pregnant women. We are also aware that younger women might have had more health care needs than is currently available in primary care. That is why we have designed a new project titled RAPID to address the health care needs of young mothers specifically. To be able to design adequate care for young mothers, it is important we learn from the experiences of care providers like you.

If you agree with what I have said so far, I will like to give you more details about your participation, and I will also require you to sign as a sign of your agreement. Also, please note that I will be recording the interview so that I am sure not to make any mistakes in what you say and to make it easier for me to check that I am accurate.

**[Facilitator to read the details on the inform consent form to each participant separately and obtain signature from will participants].**

*Predisposing factors*

**Facilitator to fill the basic demographic forms for participants who have given written consent to participants,**

Now that you have all agreed to participate, is it okay to start?

*Health beliefs.*

**1.** There are many reasons a woman attend the clinic during pregnancy. From your experience, please tell me the reasons the young girls you treated on the EXPONATE study came to the clinic when they were pregnant?

Facilitator to probe for

- - Reasons for first clinic visits
  - Reasons relating to depressive symptoms patients might have experienced during the period.

2. Do you think the treatment you delivered to young girls for depression addressed their depression?

Facilitator to probe for

- - Aspects of the intervention that patients found useful or not useful (psychoeducation, problem-solving treatment, parenting skills)
  - Care provider satisfaction with care delivered

*Enabling Factors.*

3. The next area for our discussion is keeping clinic appointments. There might be reasons that make young women want to or not want to attend clinic appointments during pregnancy. I would like to know your views on these reasons as they relate to your own experiences with adolescent mothers.

Facilitator probe

- - Reasons relating to clinic appointments for depression treatment
  - Experiences with care and interactions with service and clinic staff

4. I will like to know how much support you think young girls have during pregnancies in relation to clinic attendance for their depression.

Facilitator probe

- - Emotional and instrumental and informational support from family members, social environment
  - Support from clinic staff

*Quality of care and structural support in primary care*

5. Did you experience any specific difficulties in the treatment you delivered to adolescent mothers that were peculiar to this group?

Facilitator probe

- - Treatment related to aspects of EXPONATE care

6. Did you think adolescent mother require any need for additional support? (from other care providers) and how available was this support?

Probe for

- - Young mothers interactions with the clinic staff during receipt of treatment for depression
  - Probe for positive and negative results of such interactions

*Health behavior.*

7. The next question is about health behaviours of young mother during pregnancy.

Probe for

- - Known physical exercise adolescent mothers
  - Consumption of healthy and adequate diet by adolescent mothers
  - Time for adequate rest and sleep

*Outcome following the receipt of care.*

8. I will like to know how satisfied you were with the care you delivered to adolescent mothers with depression during the trial. I am interested in knowing how you think your interactions with them during care might have affected your recovery
